# A New Mode of Treating Exposed Dental Nerves

**Published:** 1852-10

**Authors:** 


### THE REGISTER.

The present number has been delayed beyond the usual time, which we hope will not be the case soon again. This has been owing to new arrangements for publishing, and pressing engagements of other matters which needed our close attention for the last few weeks.

We would be glad if our subscribers would more generally adopthe system of advance payment. They thereby would save the postage, and we should be far better enabled to conduct it to their satisfaction. We are satisfied, that it is with most nothing but neglect that it is not done.

We have on hand a few set of the back numbers which will we furnish to new subscribers, who will pay for volume six, in advance, at the rate of $1,50, per volume.

